# Editorial: Blood-based cellular and molecular biomarkers in acute ischemic stroke and hemorrhagic stroke, volume II

**DOI:** 10.3389/fneur.2025.1642263

**Published:** 2025-07-02

**Authors:** Ramona Schuppner, Robert G. Kowalski, Joachim E. Weber, Timo Uphaus, Gerrit M. Grosse

**Affiliations:** ^1^Department of Neurology, Hannover Medical School, Hannover, Germany; ^2^Department of Neurosurgery, Anschutz Medical Campus, University of Colorado, Aurora, CO, United States; ^3^Department of Neurology, Anschutz Medical Campus, University of Colorado, Aurora, CO, United States; ^4^Department of Neurology, Charité - Universitätsmedizin Berlin, corporate member of Freie Universität Berlin and Humboldt-Universität zu Berlin, Berlin, Germany; ^5^Department of Neurology, University Medical Centre, Johannes Gutenberg University Mainz, Mainz, Germany; ^6^Department of Neurology, University Hospital Basel, Basel, Switzerland

**Keywords:** stroke, biomarkers, personalized medicine, prognosis, prediction

Stroke remains a leading cause of death and disability worldwide, with acute ischemic and hemorrhagic strokes entailing specific diagnostic and therapeutic challenges. The search for robust biomarkers and a better understanding of disease mechanisms is crucial to improve risk prediction, acute treatment and long-term outcomes. This collection of articles in Frontiers in Neurology is the 2nd volume following the highly successful first edition and brings together original research and perspectives covering the spectrum from population-based risk assessment to molecular and genetic mechanisms underlying stroke subtypes, complications and prognosis.

A particular focus of the submitted contributions is on the differentiation of hemorrhagic and ischemic strokes, prognostication after stroke, and prediction of complications. The original papers and reviews cover inflammatory, metabolic and tissue damage markers that are promising for future clinical application.

## Inflammatory biomarkers

Systemic inflammatory processes are crucial in the pathophysiology of ischemic stroke. The immune response following stroke, particularly in large-vessel occlusion (LVO), is explored by Ma et al.. Their work reveals that AIS due to LVO rapidly induces peripheral immune activation, characterized by increased neutrophil-to-lymphocyte ratio and shifts in T-cell subsets. High-throughput sequencing of T-cell receptors further uncovers unique repertoire changes, pointing to potential diagnostic biomarkers and new insights into stroke pathophysiology.

Xue et al. analyze the Systemic Immune-Inflammation Index (SII) in over 28,000 adults from the NHANES cohort, finding a strong positive association between SII and stroke risk, independent of traditional confounding risk factors. This underscores the value of composite inflammatory indices for population-level risk stratification.

At the intersection of inflammation and metabolism, Chen J. et al. investigated the Hemoglobin, Albumin, Lymphocyte, and Platelet (HALP) score as a predictor of hemorrhagic transformation after intravenous thrombolysis in acute ischemic stroke patients. Their results show that lower HALP scores are strongly associated with both the risk and severity of hemorrhagic transformation, suggesting that this composite biomarker could guide risk stratification and treatment monitoring.

## Metabolic biomarkers

Prognostic biomarkers are essential for guiding clinical decision-making. Vasile et al. review the role of copeptin, a stable peptide derived from vasopressin, in acute ischemic stroke. Their systematic overview of the current evidence confirms that copeptin levels on admission predict both short- and long-term outcomes, as well as the efficacy of revascularization therapies, supporting its integration into prognostic models.

Recent advances in epidemiological and genetic research have identified novel risk factors and biomarker candidates for stroke. In a large-scale, 10-year prospective cohort study, Li et al. evaluated the Triglyceride-Glucose (TyG) index as a predictor of stroke incidence in a Chinese population. Their findings demonstrate that a higher TyG index, reflecting insulin resistance, is independently associated with increased risk of total and ischemic stroke, but not hemorrhagic stroke, underscoring the importance of metabolic health in stroke prevention.

Arginine derivatives are considered biomarkers of endothelial (dys-)function. Pihlasviita et al. describe plasma symmetric dimethylarginine (SDMA) as a metabolite biomarker that distinguishes severe acute ischemic stroke from hemorrhagic stroke within the first 90 min after symptom onset. Elevated SDMA levels were also linked to cardioembolic stroke and poor outcomes, highlighting its promise in diagnostic algorithms and tailored management.

Zhang et al. use Mendelian randomization to reveal that specific plasma lipids with different fatty acid side chains have causal relationships with intracerebral and subarachnoid hemorrhages. Lipids containing arachidonic acid chains therefore could be protective, while those with linoleic acid chains increase risk, offering new mechanistic insights and potential therapeutic targets.

Finally, Chen H. et al. employ a comprehensive genetic approach to explore the causality between lipidomic and immune cell profiles and ischemic stroke subtypes. Their analysis identifies genetic links between specific lipids, immune cell phenotypes, and large artery, small vessel, and cardioembolic stroke. Mediation analyses highlight the role of immune cells in the lipid–stroke pathway, suggesting new avenues for personalized prevention and intervention.

## Tissue damage biomarkers

Quality assurance in acute stroke interventions is addressed by Lieschke and Foerch, who propose serum S100B as a surrogate marker for astroglial tissue damage after mechanical thrombectomy. The authors discuss how S100B levels measured post-intervention correlate with infarct size and functional outcome, offering a potential objective metric for benchmarking endovascular therapy success.

Similarly, Freitas et al. demonstrate that neuron-specific enolase (NSE) measured 48 h after reperfusion therapy is strongly associated with 90-day functional outcomes in ischemic stroke patients. Higher NSE levels correlate with worse neurological disability, suggesting its utility as a prognostic tool for patient stratification.

Distinguishing hemorrhagic from ischemic strokes is still not possible without imaging, but would have enormous relevance for faster process times in stroke therapy. Paul et al. show that serum glial fibrillary acidic protein (GFAP) is markedly elevated in intracerebral hemorrhage compared to ischemic stroke, even in the hyperacute phase. GFAP levels also reflect the extent of tissue injury and time from onset, supporting its use in early subtype differentiation and assessment of tissue damage.

## Conclusion

Taken, the work collected in this Research Topic represents a significant step forward in the quest for reliable blood-based biomarkers in stroke. From GFAP and S100B to copeptin, NSE, and beyond, these investigations illuminate new possibilities for faster diagnosis, more accurate prognosis, and individualized patient care. As the field continues to advance, the integration of these biomarkers into clinical practice holds the promise of transforming stroke management and improving outcomes for patients worldwide. However, despite the exciting progress reflected in these studies, challenges remain. A considerable obstacle in biomarker studies often remains the limited statistical power and lack of external validation. Therefore, all efforts toward harmonization in cross-center projects are essential to advance the field.

We thank all contributing authors for their rigorous and innovative work and hope this Research Topic will inspire further research and translation into clinical practice.

